# {μ-6,6′-Dimeth­oxy-2,2′-[cyclo­hexane-1,2-diylbis(nitrilo­methyl­idyne)]diphenolato}methanol-μ-nitrato-dinitratocopper(II)europium(III)

**DOI:** 10.1107/S1600536810039103

**Published:** 2010-10-09

**Authors:** Yan Bao, Guang-Ming Li, Fan Yang, Peng-Fei Yan, Peng Chen

**Affiliations:** a School of Chemistry and Materials Science, Heilongjiang University, Harbin 150080, People’s Republic of China

## Abstract

In the title dinuclear salen-type complex, [CuEu(C_22_H_24_N_2_O_4_)(NO_3_)_3_(CH_3_OH)], the Cu^II^ ion is five-coordinated to two imine N atoms and two phenolate O atoms and one O from the bridging nitrate group. The Eu^III^ ion is ligated to three nitrate groups, four O atoms from the salen-type ligand and one methanol mol­ecule, leading to a distorted tenfold coordination for the rare earth cation. One of the three nitrate anions is disordered over two positions in a 0.66 (5):0.34 (5) ratio.

## Related literature

For the synthesis of the ligand, see: Aslantaş *et al.* (2007 ▶); Mohamed *et al.* (2003 ▶). For similar copper lanthanide complexes with a similar salen-like ligand, see: Costes *et al.* (2000 ▶, 2008 ▶); Koner *et al.* (2005 ▶); Sun *et al.* (2009 ▶).
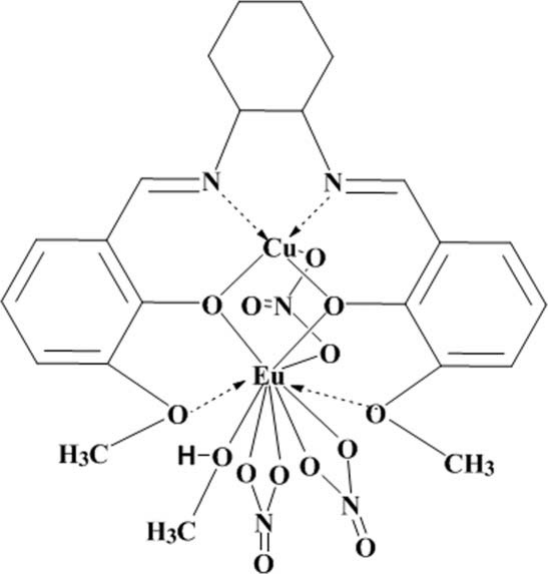


## Experimental

### 

#### Crystal data

[CuEu(C_22_H_24_N_2_O_4_)(NO_3_)_3_(CH_4_O)]
*M* *_r_* = 814.02
Monoclinic, 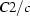

*a* = 29.305 (6) Å
*b* = 14.233 (3) Å
*c* = 14.141 (3) Å
β = 103.36 (3)°
*V* = 5739 (2) Å^3^
*Z* = 8
Mo *K*α radiation
μ = 2.99 mm^−1^
*T* = 295 K
0.14 × 0.12 × 0.11 mm

#### Data collection

Bruker SMART1000 CCD diffractometer
Absorption correction: multi-scan (*SADABS*; Sheldrick, 2003 ▶) *T*_min_ = 0.677, *T*_max_ = 0.727
27154 measured reflections
6537 independent reflections
4621 reflections with *I* > 2σ(*I*)
*R*_int_ = 0.061

#### Refinement

*R*[*F* ^2^ > 2σ(*F* ^2^)] = 0.049
*wR*(*F* ^2^) = 0.091
*S* = 1.07
6537 reflections
420 parameters
13 restraints
H-atom parameters constrained
Δρ_max_ = 0.80 e Å^−3^
Δρ_min_ = −1.11 e Å^−3^

### 

Data collection: *SMART* (Bruker, 2001 ▶); cell refinement: *SAINT-Plus* (Bruker, 2003 ▶); data reduction: *SAINT-Plus*; program(s) used to solve structure: *SHELXS97* (Sheldrick, 2008 ▶); program(s) used to refine structure: *SHELXL97* (Sheldrick, 2008 ▶); molecular graphics: *SHELXTL* (Sheldrick, 2008 ▶); software used to prepare material for publication: *SHELXL97*.

## Supplementary Material

Crystal structure: contains datablocks global, I. DOI: 10.1107/S1600536810039103/rk2221sup1.cif

Structure factors: contains datablocks I. DOI: 10.1107/S1600536810039103/rk2221Isup2.hkl

Additional supplementary materials:  crystallographic information; 3D view; checkCIF report

## Figures and Tables

**Fig. 1.**
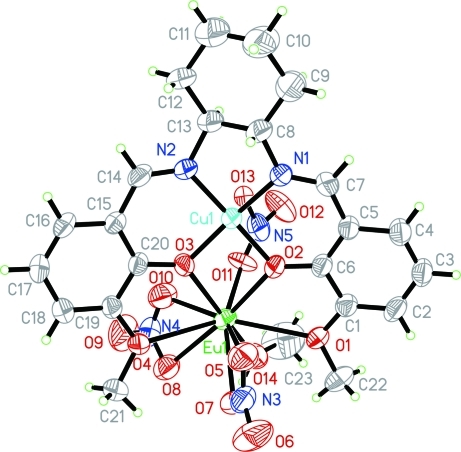
The molecular structure of the title compound with the atom numbering scheme. Displacement ellipsoids are drawn at 50% probability level. H atoms are presented as a small spheres of arbitrary radius. Only minor fragment is presented.

## supplementary crystallographic information

### Comment

In continuation of our studies of salen-type lanthanide complexes (Aslantaş *et al.*, 2007, Mohamed *et al.*, 2003, Sun *et al.*, 2009), we present here the crystal structure of the title compound. The Eu^III^ center is ligated to two bidentate nitrate groups and four oxygen atoms from the ligand, one oxygen, from the bridging nitrate group and one methanol molecule (Fig. 1). It is similar to the previously reported structures (Costes *et al.*, 2000, 2008; Koner *et al.*, 2005). The decacoordinated Eu^III^ ion presents a narrow spread in Eu–O bond distances 2.338 (18)-2.786 (4)Å. The Cu(II) ion is five-coordinated by two imine nitrogen atoms, two phenol oxygen atoms from the imine-phenolate ligand and one oxygen atom from the bridgin nitrate group.

### Experimental

To a 2:3 *Me*OH/*Me*CN solution (35 ml) of [(H_2_*L*)Eu(NO_3_)_3_] (0.2253 g, 0.3 mmol) was added an aqueous solution (10 ml) of Cu(O*Ac*)_2_˙H_2_O (0.0597 g, 0.3 mmol) at ambient temperature. After stirring for 5 hrs, the solution was filtered to remove the suspended particles. Red single crystals suitable for X-ray determination were obtained by slow diffusion of diethylether into the filtrate in one week. For CuEu(C_22_H_24_N_2_O_4_)(NO_3_)_3_CH_3_OH elemental anal. - Calc.: for C_23_H_28_N_5_O_14_EuCu: C, 33.93; H, 3.47; N, 8.60 wt%, Found: C, 33.85; H, 3.55; N, 8.58 wt%.

### Refinement

H atoms bound to C atoms were placed in calculated positions and treated as riding on their parent atoms, with C–H = 0.93Å (aromatic C), C–H = 0.97Å (methylene C), and with *U*_iso_(H) = 1.2U_eq_(C) or C–H = 0.96Å (methly C) and with *U*_iso_(H) = 1.5U_eq_(C). H atom bound to O atom was found from the Fourier difference map, the O–H distance was fixed, *U*_iso_ value is refined isotropically.

### Crystal data

**Table:**

[CuEu(C_22_H_24_N_2_O_4_)(NO_3_)_3_(CH_4_O)]	*F*(000) = 3240
*M**_r_* = 814.02	*D*_x_ = 1.884 Mg m^−^^3^
Monoclinic, *C*2/*c*	Mo *K*α radiation, λ = 0.71073 Å
Hall symbol: -C 2yc	Cell parameters from 5035 reflections
*a* = 29.305 (6) Å	θ = 3.2–27.5°
*b* = 14.233 (3) Å	µ = 2.99 mm^−^^1^
*c* = 14.141 (3) Å	*T* = 295 K
β = 103.36 (3)°	Block, red
*V* = 5739 (2) Å^3^	0.14 × 0.12 × 0.11 mm
*Z* = 8	

### Data collection

**Table:**

Bruker SMART1000 CCD diffractometer	6537 independent reflections
Radiation source: fine-focus sealed tube	4621 reflections with *I* > 2σ(*I*)
graphite	*R*_int_ = 0.061
φ and ω scans	θ_max_ = 27.5°, θ_min_ = 3.2°
Absorption correction: multi-scan (*SADABS*; Sheldrick, 2003)	*h* = −37→37
*T*_min_ = 0.677, *T*_max_ = 0.727	*k* = −18→18
27154 measured reflections	*l* = −15→18

### Refinement

**Table:**

Refinement on *F*^2^	Primary atom site location: structure-invariant direct methods
Least-squares matrix: full	Secondary atom site location: difference Fourier map
*R*[*F*^2^ > 2σ(*F*^2^)] = 0.049	Hydrogen site location: inferred from neighbouring sites
*wR*(*F*^2^) = 0.091	H-atom parameters constrained
*S* = 1.07	*w* = 1/[σ^2^(*F*_o_^2^) + (0.0119*P*)^2^ + 37.7609*P*] where *P* = (*F*_o_^2^ + 2*F*_c_^2^)/3
6537 reflections	(Δ/σ)_max_ = 0.001
420 parameters	Δρ_max_ = 0.80 e Å^−^^3^
13 restraints	Δρ_min_ = −1.11 e Å^−^^3^

### Special details

**Table:**

Geometry. All s.u.'s (except the s.u. in the dihedral angle between two l.s. planes) are estimated using the full covariance matrix. The cell s.u.'s are taken into account individually in the estimation of s.u.'s in distances, angles and torsion angles; correlations between s.u.'s in cell parameters are only used when they are defined by crystal symmetry. An approximate (isotropic) treatment of cell s.u.'s is used for estimating s.u.'s involving l.s. planes.
Refinement. Refinement of *F*^2^ against ALL reflections. The weighted *R*-factor w*R* and goodness of fit *S* are based on *F*^2^, conventional *R*-factors *R* are based on *F*, with *F* set to zero for negative *F*^2^. The threshold expression of *F*^2^ > 2σ(*F*^2^) is used only for calculating *R*-factors(gt) etc. and is not relevant to the choice of reflections for refinement. *R*-factors based on *F*^2^ are statistically about twice as large as those based on *F*, and *R*-factors based on ALL data will be even larger.

### Fractional atomic coordinates and isotropic or equivalent isotropic displacement parameters (Å^2^)

**Table:**

	*x*	*y*	*z*	*U*_iso_*/*U*_eq_	Occ. (<1)
C1	0.3809 (2)	0.1512 (4)	1.0213 (4)	0.0473 (14)	
C2	0.3876 (2)	0.1293 (4)	1.1185 (4)	0.0547 (16)	
H2	0.4176	0.1285	1.1584	0.066*	
C3	0.3492 (2)	0.1086 (4)	1.1562 (4)	0.0584 (16)	
H3	0.3537	0.0939	1.2218	0.070*	
C4	0.3048 (2)	0.1093 (4)	1.0985 (4)	0.0532 (15)	
H4	0.2795	0.0954	1.1253	0.064*	
C5	0.2970 (2)	0.1309 (4)	0.9987 (4)	0.0441 (13)	
C6	0.33567 (19)	0.1511 (4)	0.9591 (4)	0.0436 (13)	
C7	0.2496 (2)	0.1251 (4)	0.9423 (4)	0.0537 (15)	
H7	0.2265	0.1118	0.9759	0.064*	
C8	0.1873 (2)	0.1153 (7)	0.7934 (5)	0.083 (2)	
H8	0.1880	0.0502	0.7713	0.099*	
C9	0.1494 (2)	0.1200 (6)	0.8445 (5)	0.082 (2)	
H9B	0.1488	0.1819	0.8728	0.099*	
H9A	0.1549	0.0742	0.8967	0.099*	
C10	0.1024 (3)	0.1002 (7)	0.7742 (6)	0.107 (3)	
H10B	0.1015	0.0348	0.7545	0.128*	
H10A	0.0774	0.1098	0.8079	0.128*	
C11	0.0934 (3)	0.1604 (7)	0.6856 (6)	0.101 (3)	
H11B	0.0643	0.1411	0.6419	0.121*	
H11A	0.0898	0.2252	0.7040	0.121*	
C12	0.1320 (2)	0.1540 (6)	0.6347 (5)	0.080 (2)	
H12B	0.1263	0.1980	0.5808	0.096*	
H12A	0.1327	0.0912	0.6084	0.096*	
C13	0.1794 (2)	0.1757 (6)	0.7026 (5)	0.0666 (19)	
H13	0.1787	0.2414	0.7230	0.080*	
C14	0.21981 (19)	0.1500 (4)	0.5736 (4)	0.0474 (14)	
H14	0.1905	0.1488	0.5308	0.057*	
C15	0.26093 (18)	0.1363 (3)	0.5334 (4)	0.0385 (12)	
C16	0.25278 (19)	0.1125 (4)	0.4346 (4)	0.0460 (13)	
H16	0.2222	0.1086	0.3976	0.055*	
C17	0.2893 (2)	0.0952 (4)	0.3927 (4)	0.0509 (15)	
H17	0.2832	0.0795	0.3271	0.061*	
C18	0.3357 (2)	0.1004 (4)	0.4462 (4)	0.0490 (14)	
H18	0.3604	0.0866	0.4175	0.059*	
C19	0.34390 (18)	0.1265 (4)	0.5425 (4)	0.0403 (12)	
C20	0.30715 (18)	0.1460 (4)	0.5875 (4)	0.0386 (12)	
C21	0.4269 (2)	0.1079 (5)	0.5650 (4)	0.0580 (16)	
H21C	0.4224	0.0446	0.5411	0.087*	
H21B	0.4552	0.1113	0.6151	0.087*	
H21A	0.4292	0.1493	0.5128	0.087*	
C22	0.4637 (2)	0.1721 (6)	1.0336 (5)	0.078 (2)	
H22C	0.4670	0.2154	1.0868	0.117*	
H22B	0.4849	0.1891	0.9940	0.117*	
H22A	0.4707	0.1097	1.0584	0.117*	
Cu1	0.27684 (2)	0.16473 (5)	0.76583 (5)	0.04484 (17)	
Eu1	0.391175 (10)	0.21945 (2)	0.78342 (2)	0.04713 (10)	
N1	0.23598 (16)	0.1366 (4)	0.8502 (3)	0.0517 (12)	
N2	0.22134 (15)	0.1633 (3)	0.6631 (3)	0.0479 (11)	
N3	0.4412 (2)	0.0431 (4)	0.8185 (4)	0.0630 (14)	
N4	0.4195 (2)	0.3467 (4)	0.6366 (4)	0.0655 (15)	
N5	0.3249 (2)	0.3768 (4)	0.8509 (4)	0.0585 (14)	
O1	0.41630 (13)	0.1752 (3)	0.9760 (3)	0.0545 (10)	
O2	0.33298 (13)	0.1674 (3)	0.8659 (3)	0.0512 (10)	
O3	0.31885 (12)	0.1711 (3)	0.6814 (2)	0.0460 (9)	
O4	0.38772 (12)	0.1352 (3)	0.6041 (3)	0.0486 (9)	
O5	0.39729 (16)	0.0448 (3)	0.8079 (3)	0.0659 (12)	
O6	0.4629 (2)	−0.0297 (4)	0.8364 (4)	0.1043 (19)	
O7	0.46107 (14)	0.1212 (3)	0.8105 (3)	0.0603 (11)	
O8	0.44971 (15)	0.2885 (3)	0.6778 (3)	0.0654 (11)	
O9	0.4283 (2)	0.4068 (4)	0.5823 (4)	0.109 (2)	
O10	0.37998 (17)	0.3414 (3)	0.6562 (3)	0.0713 (13)	
O11'	0.3692 (5)	0.3500 (9)	0.882 (2)	0.087 (5)	0.66 (5)
O11	0.3575 (9)	0.3580 (12)	0.823 (3)	0.052 (8)	0.34 (5)
O12	0.31496 (19)	0.4366 (4)	0.9023 (4)	0.0886 (17)	
O13'	0.3033 (13)	0.3411 (8)	0.7776 (11)	0.102 (9)	0.66 (5)
O13	0.2839 (6)	0.3421 (16)	0.806 (2)	0.056 (6)	0.34 (5)
O14	0.45773 (15)	0.3208 (3)	0.8693 (3)	0.0666 (12)	
H14O	0.4821	0.3055	0.8486	0.08 (2)*	
C23	0.4620 (4)	0.4001 (7)	0.9424 (8)	0.145 (4)	
H23C	0.4639	0.4590	0.9104	0.218*	
H23B	0.4898	0.3913	0.9930	0.218*	
H23A	0.4350	0.4003	0.9700	0.218*	

### Atomic displacement parameters (Å^2^)

**Table:**

	*U*^11^	*U*^22^	*U*^33^	*U*^12^	*U*^13^	*U*^23^
C1	0.048 (3)	0.053 (3)	0.038 (3)	0.008 (3)	0.004 (3)	−0.005 (3)
C2	0.060 (4)	0.060 (4)	0.036 (3)	0.017 (3)	−0.006 (3)	−0.004 (3)
C3	0.075 (5)	0.065 (4)	0.034 (3)	0.017 (3)	0.011 (3)	0.004 (3)
C4	0.063 (4)	0.058 (4)	0.040 (3)	0.005 (3)	0.015 (3)	0.002 (3)
C5	0.048 (3)	0.049 (3)	0.034 (3)	0.005 (3)	0.008 (2)	0.001 (2)
C6	0.047 (3)	0.048 (3)	0.033 (3)	0.007 (3)	0.003 (2)	−0.001 (2)
C7	0.051 (4)	0.068 (4)	0.047 (4)	0.008 (3)	0.020 (3)	0.013 (3)
C8	0.035 (4)	0.154 (8)	0.055 (4)	0.007 (4)	0.005 (3)	0.027 (5)
C9	0.064 (5)	0.111 (6)	0.071 (5)	−0.007 (4)	0.013 (4)	0.019 (4)
C10	0.055 (5)	0.157 (9)	0.104 (7)	−0.025 (5)	0.011 (5)	0.050 (6)
C11	0.056 (5)	0.154 (9)	0.090 (6)	−0.005 (5)	0.011 (4)	0.014 (6)
C12	0.033 (3)	0.139 (7)	0.066 (5)	0.015 (4)	0.004 (3)	0.013 (4)
C13	0.035 (3)	0.109 (6)	0.056 (4)	0.005 (3)	0.010 (3)	0.017 (4)
C14	0.035 (3)	0.058 (4)	0.044 (3)	−0.002 (3)	−0.004 (2)	0.003 (3)
C15	0.037 (3)	0.040 (3)	0.035 (3)	0.001 (2)	0.003 (2)	0.005 (2)
C16	0.041 (3)	0.053 (3)	0.037 (3)	−0.004 (3)	−0.006 (2)	0.002 (2)
C17	0.060 (4)	0.060 (4)	0.031 (3)	−0.002 (3)	0.007 (3)	0.000 (3)
C18	0.050 (3)	0.059 (4)	0.040 (3)	0.001 (3)	0.014 (3)	−0.002 (3)
C19	0.039 (3)	0.049 (3)	0.033 (3)	−0.003 (2)	0.010 (2)	0.002 (2)
C20	0.040 (3)	0.043 (3)	0.031 (3)	−0.001 (2)	0.005 (2)	0.006 (2)
C21	0.042 (3)	0.079 (5)	0.055 (4)	0.008 (3)	0.016 (3)	−0.002 (3)
C22	0.045 (4)	0.108 (6)	0.067 (5)	0.008 (4)	−0.014 (3)	0.003 (4)
Cu1	0.0323 (3)	0.0680 (5)	0.0333 (4)	0.0023 (3)	0.0059 (3)	0.0017 (3)
Eu1	0.03248 (14)	0.06129 (19)	0.04366 (16)	−0.00211 (15)	0.00070 (11)	−0.00113 (15)
N1	0.037 (3)	0.077 (4)	0.041 (3)	0.005 (2)	0.009 (2)	0.014 (2)
N2	0.030 (2)	0.069 (3)	0.042 (3)	0.001 (2)	0.003 (2)	0.003 (2)
N3	0.066 (4)	0.070 (4)	0.047 (3)	0.017 (3)	0.000 (3)	0.001 (3)
N4	0.074 (4)	0.074 (4)	0.045 (3)	−0.010 (3)	0.005 (3)	0.004 (3)
N5	0.077 (4)	0.053 (3)	0.053 (4)	−0.003 (3)	0.028 (3)	0.004 (3)
O1	0.037 (2)	0.081 (3)	0.040 (2)	0.007 (2)	−0.0019 (18)	0.0002 (19)
O2	0.039 (2)	0.078 (3)	0.034 (2)	0.003 (2)	0.0021 (17)	0.0062 (19)
O3	0.034 (2)	0.070 (3)	0.033 (2)	−0.0068 (18)	0.0078 (16)	−0.0075 (18)
O4	0.032 (2)	0.073 (3)	0.040 (2)	0.0018 (18)	0.0079 (17)	−0.0034 (19)
O5	0.052 (3)	0.072 (3)	0.068 (3)	−0.008 (2)	0.004 (2)	−0.003 (2)
O6	0.098 (4)	0.082 (4)	0.121 (5)	0.035 (3)	0.000 (4)	0.017 (3)
O7	0.040 (2)	0.077 (3)	0.060 (3)	0.002 (2)	0.005 (2)	0.004 (2)
O8	0.052 (3)	0.078 (3)	0.062 (3)	0.000 (2)	0.003 (2)	0.004 (2)
O9	0.117 (5)	0.120 (5)	0.090 (4)	−0.014 (4)	0.025 (4)	0.041 (4)
O10	0.057 (3)	0.077 (3)	0.078 (3)	0.001 (2)	0.011 (3)	0.011 (3)
O11'	0.059 (6)	0.086 (7)	0.114 (16)	0.011 (5)	0.019 (8)	−0.003 (7)
O11	0.038 (11)	0.047 (8)	0.082 (18)	−0.001 (6)	0.034 (11)	−0.008 (9)
O12	0.111 (4)	0.077 (3)	0.098 (4)	−0.006 (3)	0.065 (4)	−0.024 (3)
O13'	0.19 (2)	0.064 (6)	0.036 (6)	0.013 (8)	−0.014 (9)	0.001 (4)
O13	0.045 (10)	0.091 (11)	0.034 (11)	0.017 (7)	0.011 (6)	0.005 (8)
O14	0.049 (3)	0.083 (3)	0.067 (3)	−0.015 (2)	0.013 (2)	−0.022 (2)
C23	0.121 (9)	0.118 (8)	0.174 (11)	−0.018 (7)	−0.012 (8)	−0.056 (8)

### Geometric parameters (Å, °)

**Table:**

C1—C2	1.378 (8)	C20—O3	1.340 (6)
C1—O1	1.383 (7)	C21—O4	1.437 (6)
C1—C6	1.411 (7)	C21—H21C	0.9600
C2—C3	1.383 (8)	C21—H21B	0.9600
C2—H2	0.9300	C21—H21A	0.9600
C3—C4	1.366 (8)	C22—O1	1.439 (7)
C3—H3	0.9300	C22—H22C	0.9600
C4—C5	1.411 (7)	C22—H22B	0.9600
C4—H4	0.9300	C22—H22A	0.9600
C5—C6	1.404 (7)	Cu1—O3	1.905 (3)
C5—C7	1.436 (8)	Cu1—O2	1.906 (4)
C6—O2	1.323 (6)	Cu1—N2	1.914 (4)
C7—N1	1.282 (7)	Cu1—N1	1.917 (5)
C7—H7	0.9300	Cu1—Eu1	3.3927 (10)
C8—C9	1.458 (9)	Eu1—O11	2.330 (17)
C8—N1	1.497 (8)	Eu1—O3	2.374 (3)
C8—C13	1.518 (9)	Eu1—O2	2.395 (4)
C8—H8	0.9800	Eu1—O7	2.436 (4)
C9—C10	1.528 (10)	Eu1—O10	2.467 (5)
C9—H9B	0.9700	Eu1—O11'	2.494 (18)
C9—H9A	0.9700	Eu1—O14	2.503 (4)
C10—C11	1.490 (10)	Eu1—O5	2.511 (5)
C10—H10B	0.9700	Eu1—O8	2.706 (4)
C10—H10A	0.9700	Eu1—O1	2.725 (4)
C11—C12	1.478 (10)	Eu1—O4	2.785 (4)
C11—H11B	0.9700	Eu1—N3	2.891 (6)
C11—H11A	0.9700	N3—O6	1.212 (7)
C12—C13	1.525 (8)	N3—O5	1.261 (6)
C12—H12B	0.9700	N3—O7	1.271 (7)
C12—H12A	0.9700	N4—O9	1.215 (7)
C13—N2	1.475 (7)	N4—O8	1.254 (7)
C13—H13	0.9800	N4—O10	1.254 (7)
C14—N2	1.270 (7)	N5—O11	1.146 (16)
C14—C15	1.459 (7)	N5—O13'	1.196 (12)
C14—H14	0.9300	N5—O12	1.199 (7)
C15—C20	1.400 (7)	N5—O13	1.32 (2)
C15—C16	1.403 (7)	N5—O11'	1.324 (16)
C16—C17	1.360 (8)	O11'—O11	0.829 (18)
C16—H16	0.9300	O11—O13'	1.59 (2)
C17—C18	1.396 (8)	O13'—O13	0.77 (2)
C17—H17	0.9300	O14—C23	1.516 (10)
C18—C19	1.379 (7)	O14—H14O	0.8608
C18—H18	0.9300	C23—H23C	0.9600
C19—O4	1.381 (6)	C23—H23B	0.9600
C19—C20	1.399 (7)	C23—H23A	0.9600
			
C2—C1—O1	124.7 (5)	O7—Eu1—O14	73.87 (16)
C2—C1—C6	121.2 (6)	O10—Eu1—O14	84.60 (16)
O1—C1—C6	114.1 (5)	O11'—Eu1—O14	64.7 (3)
C1—C2—C3	119.4 (6)	O11—Eu1—O5	145.6 (6)
C1—C2—H2	120.3	O3—Eu1—O5	79.76 (14)
C3—C2—H2	120.3	O2—Eu1—O5	70.27 (14)
C4—C3—C2	121.0 (6)	O7—Eu1—O5	51.62 (14)
C4—C3—H3	119.5	O10—Eu1—O5	142.23 (15)
C2—C3—H3	119.5	O11'—Eu1—O5	132.8 (6)
C3—C4—C5	120.8 (6)	O14—Eu1—O5	118.75 (15)
C3—C4—H4	119.6	O11—Eu1—O8	100.6 (5)
C5—C4—H4	119.6	O3—Eu1—O8	111.12 (12)
C6—C5—C4	118.9 (5)	O2—Eu1—O8	174.02 (13)
C6—C5—C7	123.9 (5)	O7—Eu1—O8	71.23 (15)
C4—C5—C7	117.1 (5)	O10—Eu1—O8	48.42 (14)
O2—C6—C5	124.4 (5)	O11'—Eu1—O8	108.4 (4)
O2—C6—C1	116.9 (5)	O14—Eu1—O8	62.38 (14)
C5—C6—C1	118.7 (5)	O5—Eu1—O8	113.67 (15)
N1—C7—C5	126.2 (5)	O11—Eu1—O1	89.2 (10)
N1—C7—H7	116.9	O3—Eu1—O1	122.53 (12)
C5—C7—H7	116.9	O2—Eu1—O1	60.03 (12)
C9—C8—N1	117.7 (6)	O7—Eu1—O1	71.76 (13)
C9—C8—C13	114.0 (6)	O10—Eu1—O1	148.33 (15)
N1—C8—C13	106.2 (5)	O11'—Eu1—O1	70.1 (7)
C9—C8—H8	106.0	O14—Eu1—O1	69.39 (14)
N1—C8—H8	106.0	O5—Eu1—O1	68.86 (13)
C13—C8—H8	106.0	O8—Eu1—O1	125.17 (12)
C8—C9—C10	110.2 (7)	O11—Eu1—O4	130.9 (11)
C8—C9—H9B	109.6	O3—Eu1—O4	58.82 (11)
C10—C9—H9B	109.6	O2—Eu1—O4	115.45 (12)
C8—C9—H9A	109.6	O7—Eu1—O4	75.43 (13)
C10—C9—H9A	109.6	O10—Eu1—O4	70.60 (14)
H9B—C9—H9A	108.1	O11'—Eu1—O4	150.3 (7)
C11—C10—C9	113.5 (7)	O14—Eu1—O4	123.37 (13)
C11—C10—H10B	108.9	O5—Eu1—O4	71.69 (13)
C9—C10—H10B	108.9	O8—Eu1—O4	63.13 (12)
C11—C10—H10A	108.9	O1—Eu1—O4	139.01 (12)
C9—C10—H10A	108.9	O11—Eu1—N3	156.8 (10)
H10B—C10—H10A	107.7	O3—Eu1—N3	101.48 (15)
C12—C11—C10	111.5 (7)	O2—Eu1—N3	92.16 (16)
C12—C11—H11B	109.3	O7—Eu1—N3	25.85 (14)
C10—C11—H11B	109.3	O10—Eu1—N3	135.45 (16)
C12—C11—H11A	109.3	O11'—Eu1—N3	137.5 (7)
C10—C11—H11A	109.3	O14—Eu1—N3	96.30 (17)
H11B—C11—H11A	108.0	O5—Eu1—N3	25.78 (14)
C11—C12—C13	111.6 (6)	O8—Eu1—N3	92.80 (16)
C11—C12—H12B	109.3	O1—Eu1—N3	67.59 (13)
C13—C12—H12B	109.3	O4—Eu1—N3	72.12 (13)
C11—C12—H12A	109.3	C7—N1—C8	123.6 (5)
C13—C12—H12A	109.3	C7—N1—Cu1	124.5 (4)
H12B—C12—H12A	108.0	C8—N1—Cu1	111.3 (4)
N2—C13—C8	105.9 (5)	C14—N2—C13	123.7 (5)
N2—C13—C12	117.0 (6)	C14—N2—Cu1	125.9 (4)
C8—C13—C12	110.9 (6)	C13—N2—Cu1	110.4 (4)
N2—C13—H13	107.5	O6—N3—O5	120.8 (7)
C8—C13—H13	107.5	O6—N3—O7	122.5 (6)
C12—C13—H13	107.5	O5—N3—O7	116.7 (5)
N2—C14—C15	124.5 (5)	O6—N3—Eu1	177.7 (5)
N2—C14—H14	117.8	O5—N3—Eu1	60.0 (3)
C15—C14—H14	117.8	O7—N3—Eu1	56.7 (3)
C20—C15—C16	119.2 (5)	O9—N4—O8	122.0 (7)
C20—C15—C14	123.8 (5)	O9—N4—O10	121.5 (7)
C16—C15—C14	117.0 (5)	O8—N4—O10	116.5 (6)
C17—C16—C15	120.5 (5)	O9—N4—Eu1	172.0 (6)
C17—C16—H16	119.7	O8—N4—Eu1	63.9 (3)
C15—C16—H16	119.7	O10—N4—Eu1	52.8 (3)
C16—C17—C18	121.3 (5)	O11—N5—O13'	85.5 (11)
C16—C17—H17	119.4	O11—N5—O12	135.6 (13)
C18—C17—H17	119.4	O13'—N5—O12	132.2 (17)
C19—C18—C17	118.4 (5)	O11—N5—O13	119.4 (13)
C19—C18—H18	120.8	O13'—N5—O13	35.4 (10)
C17—C18—H18	120.8	O12—N5—O13	103.4 (12)
C18—C19—O4	124.9 (5)	O11—N5—O11'	38.4 (11)
C18—C19—C20	121.7 (5)	O13'—N5—O11'	116.5 (11)
O4—C19—C20	113.4 (4)	O12—N5—O11'	111.2 (12)
O3—C20—C19	117.1 (5)	O13—N5—O11'	139.9 (11)
O3—C20—C15	124.1 (5)	C1—O1—C22	117.3 (5)
C19—C20—C15	118.8 (5)	C1—O1—Eu1	117.5 (3)
O4—C21—H21C	109.5	C22—O1—Eu1	125.1 (4)
O4—C21—H21B	109.5	C6—O2—Cu1	125.4 (3)
H21C—C21—H21B	109.5	C6—O2—Eu1	130.9 (3)
O4—C21—H21A	109.5	Cu1—O2—Eu1	103.56 (16)
H21C—C21—H21A	109.5	C20—O3—Cu1	123.6 (3)
H21B—C21—H21A	109.5	C20—O3—Eu1	131.9 (3)
O1—C22—H22C	109.5	Cu1—O3—Eu1	104.37 (15)
O1—C22—H22B	109.5	C19—O4—C21	116.2 (4)
H22C—C22—H22B	109.5	C19—O4—Eu1	116.5 (3)
O1—C22—H22A	109.5	C21—O4—Eu1	127.0 (3)
H22C—C22—H22A	109.5	N3—O5—Eu1	94.2 (4)
H22B—C22—H22A	109.5	N3—O7—Eu1	97.5 (3)
O3—Cu1—O2	83.82 (15)	N4—O8—Eu1	91.6 (4)
O3—Cu1—N2	94.78 (18)	N4—O10—Eu1	103.3 (4)
O2—Cu1—N2	178.52 (19)	O11—O11'—N5	59.1 (16)
O3—Cu1—N1	170.7 (2)	O11—O11'—Eu1	69 (2)
O2—Cu1—N1	95.66 (18)	N5—O11'—Eu1	113.0 (14)
N2—Cu1—N1	85.8 (2)	O11'—O11—N5	83 (2)
O3—Cu1—Eu1	42.67 (10)	O11'—O11—O13'	122 (2)
O2—Cu1—Eu1	43.33 (11)	N5—O11—O13'	48.6 (8)
N2—Cu1—Eu1	135.19 (14)	O11'—O11—Eu1	92 (2)
N1—Cu1—Eu1	138.63 (15)	N5—O11—Eu1	135.5 (12)
O11—Eu1—O3	91.4 (8)	O13'—O11—Eu1	102.4 (15)
O11—Eu1—O2	75.9 (5)	O13—O13'—N5	81 (2)
O3—Eu1—O2	64.54 (12)	O13—O13'—O11	125 (2)
O11—Eu1—O7	146.6 (9)	N5—O13'—O11	45.9 (8)
O3—Eu1—O7	121.96 (14)	O13—O13'—Eu1	135 (2)
O2—Eu1—O7	114.39 (14)	N5—O13'—Eu1	86.7 (16)
O11—Eu1—O10	65.7 (9)	O11—O13'—Eu1	47.4 (11)
O3—Eu1—O10	79.09 (14)	O13'—O13—N5	64 (2)
O2—Eu1—O10	125.68 (14)	C23—O14—Eu1	133.8 (5)
O7—Eu1—O10	118.94 (16)	C23—O14—H14O	118.5
O11—Eu1—O11'	19.4 (5)	Eu1—O14—H14O	107.7
O3—Eu1—O11'	104.2 (4)	O14—C23—H23C	109.5
O2—Eu1—O11'	69.7 (4)	O14—C23—H23B	109.5
O7—Eu1—O11'	131.0 (6)	H23C—C23—H23B	109.5
O10—Eu1—O11'	82.8 (7)	O14—C23—H23A	109.5
O11—Eu1—O14	73.8 (7)	H23C—C23—H23A	109.5
O3—Eu1—O14	161.47 (15)	H23B—C23—H23A	109.5
O2—Eu1—O14	120.33 (14)		

## References

[bb1] Aslantaş, M., Tümer, M., Şahin, E. & Tümer, F. (2007). *Acta Cryst.* E**63**, o644–o645.

[bb2] Bruker (2001). *SMART* Bruker AXS Inc., Madison, Wisconsin, USA.

[bb3] Bruker (2003). *SAINT-Plus* Bruker AXS Inc., Madison, Wisconsin, USA.

[bb4] Costes, J. P., Dahan, F. & Dupuis, A. (2000). *Inorg. Chem.* **39**, 165–168. 10.1021/ic990865h 11272520

[bb5] Costes, J. P., Donnadieu, B., Gheorghe, R. & Tuchagues, J. P. (2008). *Eur. J. Inorg. Chem.* pp. 5235–5244.

[bb6] Koner, R., Lee, G. H., Wang, Y., Wei, H. H. & Mohanta, S. (2005). *Eur. J. Inorg. Chem.* pp. 1500–1505.

[bb7] Mohamed, E. M., Muralidharan, S., Panchanatheswaran, K., Ramesh, R., Low, J. N. & Glidewell, C. (2003). *Acta Cryst.* C**59**, o367–o369. 10.1107/s0108270103010205 12855858

[bb8] Sheldrick, G. M. (2003). *SADABS* University of Göttingen, Germany.

[bb9] Sheldrick, G. M. (2008). *Acta Cryst.* A**64**, 112–122. 10.1107/S0108767307043930 18156677

[bb10] Sun, W.-B., Yan, P.-F., Li, G.-M. & Hou, G.-F. (2009). *Acta Cryst.* E**65**, m780–m781. 10.1107/S1600536809022077 PMC2969354 21582709

